# Corticotropin-releasing factor receptor-1 modulates biomarkers of DNA oxidation in Alzheimer’s disease mice

**DOI:** 10.1371/journal.pone.0181367

**Published:** 2017-07-27

**Authors:** Cheng Zhang, Robert A. Rissman

**Affiliations:** 1 Department of Neurosciences, University of California, San Diego School of Medicine, La Jolla, California, United States of America; 2 Veterans Affairs San Diego Healthcare System, San Diego, California, United States of America; Oregon Health and Science University, UNITED STATES

## Abstract

Increased production of hydroxyl radical is the main source of oxidative damage in mammalian DNA that accumulates in Alzheimer’s disease (AD). Reactive oxygen species (ROS) react with both nuclear DNA (nDNA) and mitochondrial DNA (mtDNA) to generate 8-hydroxy-2’-deoxyguanosine (8-OHdG), both of which can be measured in the urine. Knowledge of this pathway has positioned measurement of urine 8-OHdG as a reliable index of DNA oxidation and a potential biomarker target for tracking early cellular dysfunction in AD. Furthermore, epigenetic studies demonstrate decreased global DNA methylation levels (e.g. 5-methyl-2’-deoxycytidine, 5-mdC) in AD tissues. Moreover, stress hormones can activate neuronal oxidative stress which will stimulate the release of additional stress hormones and result in damages to hippocampal neurons in the AD brain. Our previous work suggests that treating AD transgenic mice the type-1 corticotropin-releasing factor receptor (CRFR1) antagonist, R121919, to reduce stress signaling, prevented onset of cognitive impairment, synaptic/dendritic loss and Aβ plaque accumulation. Therefore, to investigate whether levels of DNA oxidation can be impacted by the same therapeutic approach, urine levels of hydrogen peroxide, 8-OHdG, 5-mdC and total antioxidant capacity (TAC) were analyzed using an AD Tg mouse model. We found that Tg animals had an 80% increase in hydrogen peroxide levels compared to wild type (Wt) counterparts, an effect that could be dramatically reversed by the chronic administration with R121919. A significant decrease of 8-OHdG levels was observed in Tg mice treated with CRFR1 antagonist. Collectively our data suggest that the beneficial effects of CRFR1 antagonism seen in Tg mice may be mechanistically linked to the modulation of oxidative stress pathways.

## Introduction

Neurodegeneration in Alzheimer’s disease (AD) is characterized by extensive synaptic and neuronal loss and two pathological markers; amyloid-beta (Aβ) plaques and neurofibrillary tangles (NFT). Our previous work suggests that oxidative stress can serve as a source of macromolecule damage early in the pathogenesis of AD [1,2,3,4]. In particular, we have found that reactive oxygen species (ROS) and hydroxyl radicals can modify the structure of nucleotides by oxidizing nitrogenous bases and deoxyribose to cause DNA backbone damage, strand breaks, cross-linking DNA to DNA or DNA to protein. DNA oxidation damage, therefore, has a series of detrimental effects on DNA mutations, RNA transcriptional impairments and protein translational deficits [5]. Moreover, a significant increase in urine levels of oxidized DNA (8-hydroxy-2’-deoxyguanosine, 8-OHdG) has been reported in AD transgenic (Tg) mice [4]. Oxidative stress may be a critical risk factor in AD neuropathology [6], involving a dysregulation of oxidative redox homeostasis and resulting in an increase of DNA oxidation. Elevated oxidative insults (hydroxyl radicals) are associated with oxidative damage of mammalian macromolecules, including RNA lesions, protein modification and lipid oxidation [5]. Because ROS can react with both nuclear DNA (nDNA) and mitochondrial DNA (mtDNA) to generate 8-OHdG, urine 8-OHdG levels are a reliable index of DNA oxidation and may be an important biomarker of AD pathology. Specifically, urine provides a better matrix to quantify 8-OHdG, because 8-OHdG can present either as a free nucleoside or bound to DNA. To minimize the risk of overestimation of 8-OHdG baseline levels due to sample complexity, urine sample are a more suitable choice compared to plasma, cell lysis and tissues [4]. Furthermore, DNA methylation is considered as a critical epigenetic change, which is associated with proliferation and differentiation. Studies have shown that a decrease of DNA methylation levels, especially reduction in global 5-methyl-2’-deoxycytidine (5-mdC) was found in aging cells from various types of samples [7]. Overall antioxidant capacity may reflect the cumulative effect of total antioxidants in cell lysates and body fluids, including plasma, serum, urine and saliva. Published reports suggest that total antioxidant capacity (TAC) in human serum offers a higher contribution in combating against free radicals than individual antioxidant, thereafter, measurement of TAC may provide an insightful explanation of the dynamic changes in ROS and antioxidant system [8].

The hippocampus is particularly vulnerable to oxidative stress and is a region with high expression of the type-1 corticotropin-releasing factor receptor (CRFR1) [9,10,11]. Elevated production and release of stress hormones may endanger hippocampal neurons (CA) in AD [9,12,13,14]. Stress hormones may aggravate neuronal oxidative stress which can further stimulate the release of stress hormones, resulting in a damaging cycle for hippocampal neurons in the AD brain [9]. For example, stress promotes the production of ROS, leading to DNA oxidative damage, and at the same time, stress is also inhibiting DNA repair systems which increase DNA lesion accumulation. Our previous work demonstrates that blocking the stress-signaling system using R121919, an antagonist to the type-1 corticotropin-releasing factor receptor (CRFR1) can prevent cognitive impairment, synaptic/dendritic loss, and Aβ plaque accumulation in AD transgenic (Tg) mice [15]. Whether modulation of the ROS system is mechanistically linked to these disease-modifying effects is currently unknown.

We hypothesize that dysregulation of hypothalamic-pituitary-adrenal (HPA) axis leads to increased release of CRF and stress steroids and subsequent to ROS overproduction and oxidative damage. We further hypothesize that DNA oxidative levels can be reduced by blocking stress signal pathway using a CRFR1 antagonist. In this study, Tg and age-matched Wt animals starting at 1-month-old were treated daily with R121919 (20 mg/kg/d, sc) for 5-months to investigate the role of CRFR1 in neuronal oxidative stress. To investigate the impact of drug treatment on DNA oxidation, urine levels of hydrogen peroxide, 8-OHdG, 5-mdC, and TAC were analyzed.

## Materials and methods

### Animals

AD Tg mice (Borchelt line 85, *APP-Swe*_*K595N*, *M596L*_ and *PS1*_*dE9*_; Jackson Laboratories, Bar Harbor, ME [4,16] that reliably accumulate Aβ plaques in the cortex and hippocampus beginning at 4–5 month of age were used in this study. At 30 days of age, male and female mice AD Tg and Wt littermates were divided into 8 cohorts (N = 5 each) to examine the impact of R121919 (20 mg/kg/d, sc) on DNA oxidation over 5 months. A total of 40 mice were used. To gain insight into TAC levels in more severe disease stage, AD Tg mice of 24 months of age were used. All mice were housed (2–4 mice/cage) in a temperature-controlled room (22°C) with a 12 h light-dark cycle. After treatment, animals were euthanized by cervical dislocation. The UCSD Institutional Mouse Care and Use Committee (IACUC) approved the study design and all experimental protocols used.

### CRFR1 antagonist treatment

**Fig 1:**
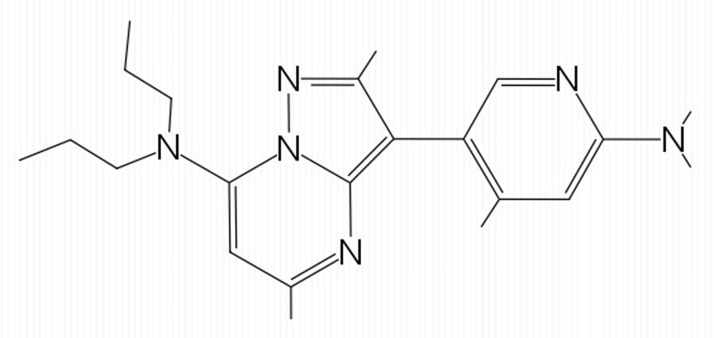
The chemical structure of CRFR1 antagonist (R121919).

R121919 is a second-generation CRFR1 antagonist that has been extensively used in animal models and in early phase human studies (Fig 1). In our previous work, 20 mg/kg/d sc proved to be the optimal dose that maximized antagonism at CRFR1 while maintaining solubility and safety [17,18,19]. Based on our radioligand binding study, R121919 was sufficiently binding the targets at 20 mg/kg/d in mouse brain [15]. R121919 was dissolved in 0.3% tartaric acid and 5% v/v polyethoxylated castor oil as described previously [18]. R121919 was a gift from Dr. Kenner Rice (NIH).

### Anxiety behavior

Open field test was used to document potential motor deficits with R121919 treatment and to investigate whether long-term treatment of R121919 can induce anxiety behavior. Anxiety level in the open field test was determined by measuring time spent and distance traveled, and observations of variously horizontal and vertical behaviors. Because rodents naturally avoid open fields and prefer to stay in the periphery of the testing cage or against walls (thigmotaxis), mice tend to show fewer center crossings during periods of stress. For this reason, the pattern of exploration in the open-field (center vs. periphery) can be used as a good measure of anxiety in rodents. Mice were placed in one of the corners of an open field box where total activity, rearings, time, distance traveled during exploration, and other movements were measured during each 5 min trial. The open field test consisted of 2 trials/day (5 min/trial) over the course of 2 consecutive days. The inter-trial interval (ITI) was 3 hours. The Kinder Motor Monitor Cage rack system with x, y, and z coordinates with a 7 x 15 beam configuration. The exact testing times were scheduled at 10 am and 1 pm on each day. Test chambers were thoroughly cleaned between runs and disinfected at the end of each day. Mice naturally prefer to stay in the periphery of the testing box instead of exploring the open fields, thereafter, the decrease of center crossings can be a sign for animals under stress. Also, that is why the pattern of exploration in the open-field (center vs. periphery) can be used as a good indication of anxiety in rodents.

### Quantification of hydrogen peroxide levels

Levels of hydrogen peroxide in the urine from our cohorts were determined using commercially available bioassay kits from Cayman Chemical Company (Ann Arbor, MI) used according to the manufacturer instructions. UrineUrine samples were collected from animal cohorts as described above and read with an iMark Microplate Reader (Bio-Rad Laboratories Inc., Hercules, CA) at 595 nm.

### Quantification of 8-OHdG levels

8-OHdG levels in the urine from our cohorts were determined using a commercially available bioassay kit from Cell Biolabs (San Diego, CA) according to the manufacturer instructions and read with an iMark Microplate Reader (Bio-Rad, Hercules, CA) at 450 nm.

### Quantification of 5-mdC content

5-mdC content in the urine was determined using a commercial assay kit from Cayman Chemical Company (Ann Arbor, MI) according to the manufacturer instructions and read with an iMark Microplate Reader (Bio-Rad, Hercules, CA) at 415 nm.

### Quantification of TAC levels

TAC was used to analyze inform on levels antioxidant levels in the cortex with an antioxidant assay kit (Cayman Chemical Company, Ann Arbor, MI), used according to the manufacturer's instructions. Cortical tissue homogenates, urine, and serum samples were diluted 1:10 with Sample Buffer prior to assaying. TAC levels were read on an iMark Plate Reader (Bio-Rad Laboratories Inc., Hercules, CA) at 750 nm.

### Statistical analyses

Statistical analyses between groups were conducted via either t-test, one-way or two-way ANOVA with Tukey's Multiple Comparison post-hoc test using GraphPad Prism 7 software (La Jolla, CA). A value of P<0.05 was accepted as significant. All values have been expressed as Mean ± SEM.

## Results

### Open field test

To confirm the viability of animals for behavioral testing, we ensured that all mice were in good general health and that motor abilities were normal. The open field test was employed to examine motor changes induced by stress in mice treated with R121919 compared to vehicle. No significant differences were found in total distance, immobile time, time spent in the periphery zone, thigmotaxis, total activity, number of rearings, time spent in the center zone, and distance travelled in the center zone regardless of the treatment, genotype, or gender, indicating that no abnormal anxiety-like behavior was induced by vehicle or R121919 treatment (Table 1).

**Table 1:** Behavioral data.

Treatment	Total Distance (m)	Immobility (s)	Time/Periphery (% of time)	Thigmotaxis (distance ratio)	Total Activity (no.)	Rearing (no.)	Time/Center (% of time)	Distance/Center (% of distance)
Vehicle	19.38±1.56	295.23±0.84	86.50±2.60	0.80±0.02	654.56±68.26	68.31±9.05	13.50±2.60	20.05±2.26
Drug	16.85±0.69	297.36±0.32	86.7±1.10	0.80±0.01	579.81±27.99	58.32±5.20	13.30±1.10	22.05±1.11

Exploratory locomotor activity, including total distance, immobile time, time spent in the periphery zone, thigmotaxis, total activity, number of rearings, time spent in the center zone, distance traveled in the center zone have been recorded and calculated for the exploratory locomotor activity test. No difference has been found in these activities regardless of the treatment, genotype, and gender, indicating no abnormal anxiety-like behavior was seen in drug-treated and non-treated animals. All data are expressed as Mean ± SEM, N = 5 mice/group.

### Hydrogen peroxide status

**Fig 2:**
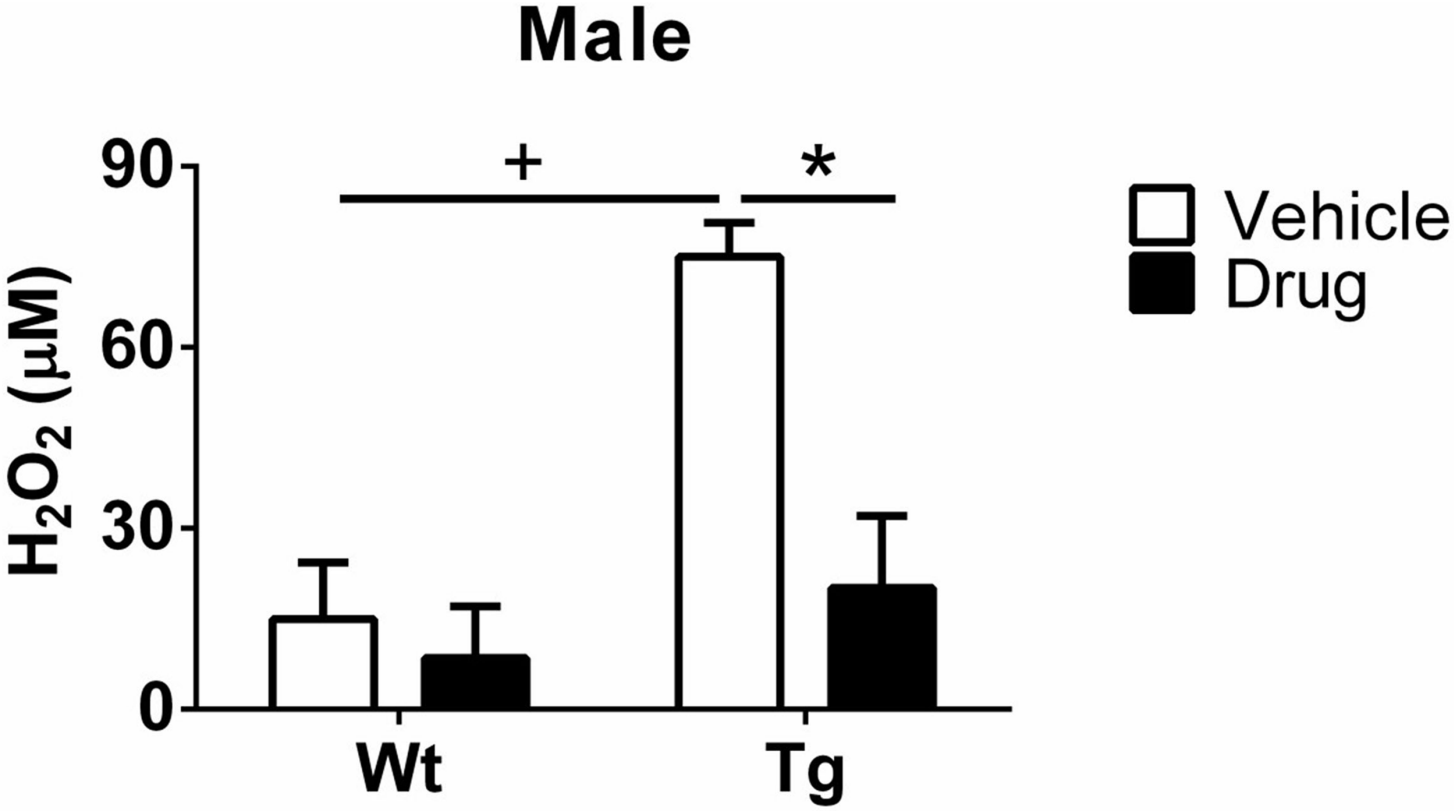
Determination of urine H_2_O_2_ levels in male groups (treatment & genotype). A significant genotype effect was found between Wt-vehicle and Tg-vehicle males, indicating a significant oxidative damage might exist in Tg males. Moreover, there was no difference between Wt-vehicle and Wt-drug males. However, a significant reduction was detected in Tg-drug males compared to their Tg-vehicle counterparts, which demonstrated that the drug might have a potentially protective effect against ROS. As for female groups, H_2_O_2_ concentrations were below the level of detection. Genotype effect (Wt v.s. Tg): +P<0.05. Treatment effect (vehicle v.s. drug): *P<0.05. All data are expressed as Mean ± SEM, N = 5 mice/group.

Hydrogen peroxide (H_2_O_2_) levels are an important index of oxidative insults and a sensitive index for assessing levels of reactive oxygen species (ROS). Urine H_2_O_2_ levels from each group were determined by ELISA (Cayman Chemical, MI). As seen in Fig 2, a significant genotype effect was found between Wt-vehicle and Tg-vehicle males, which suggests that oxidative damage exists in male Tg mice. Moreover, there was no difference between Wt-vehicle and Wt-drug males. However, a significant reduction was found in Tg-drug males compared to Tg-vehicle counterparts, implicating a beneficial effect of R121919 on ROS production. In female groups, H_2_O_2_ concentrations fell below the level of detection. All data are expressed as Mean ± SEM, N = 5 mice/group. Genotype effect (Wt v.s. Tg): +P<0.05. Treatment effect (vehicle v.s. drug): *P<0.05.

### 8-OHdG levels

**Fig 3:**
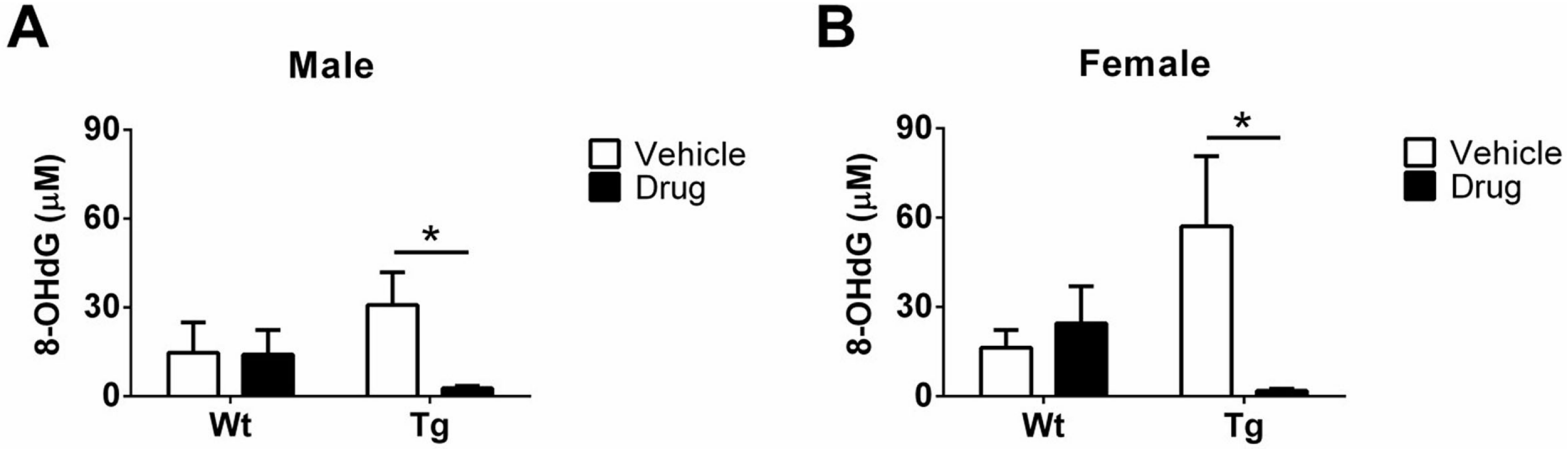
Quantification of urine 8-OHdG levels in different groups by the ELISA kit. (A) 8-OHdG levels of male groups. No difference was found in Wt-males regardless of treatment, while a significant decrease of 8-OHdG levels was observed in Tg-males treated with the drug compared to their Tg-vehicle littermates. (B) 8-OHdG levels of female groups. Wt-females had no difference regardless of treatment, however, Tg-females treated with drug showed a significant reduction of 8-OHdG levels. There was no genotype effect in both males and females. Treatment effect (vehicle v.s. drug): *P<0.05. All data are expressed as Mean ± SEM, N = 5 mice/group.

8-OHdG is a sensitive biomarker of oxidative stress. To explore whether R121919 can protect against oxidative stress, urine 8-OHdG levels of each group were examined. In terms of genotype effects, we found no difference in either male or female mice. As seen in Fig 3A, no significance was found in Wt-males regardless of treatment, while a significant decrease of 8-OHdG levels was observed in Tg-males treated with R121919 compared to Tg-vehicle cohorts (*P<0.05). Likewise, Wt-females had no change in 8-OHdG regardless of treatment, however, Tg-females treated with drug displayed a significant reduction of 8-OHdG levels (*P<0.05) (Fig 3B).

### 5-mdC content

5-mdC is a marker of DNA methylation. 5-mdC levels in the urine were examined by ELISA. No statistically significant differences were seen regardless of treatment, genotype or gender effect (Table 2).

**Table 2:** Measurement of urine 5-mdC levels under different conditions.

5-mdC (μM)	Wt-Vehicle	Wt-Drug	Tg-Vehicle	Tg-Drug
Male	17.06 ± 1.97	17.81 ± 0.54	17.11 ± 0.45	16.64 ± 1.44
Female	17.76 ± 0.23	17.50 ± 1.90	16.36 ± 1.52	16.43 ± 1.13

5-mdC levels of male groups: No significance was found regardless of treatment or genotype. 5-mdC levels of female groups: Wt-females had no significant difference regardless of treatment or genotype. No gender effect was found in urine 5-mdC levels among these animals. All data are expressed as Mean ± SEM, N = 5 mice/group.

### TAC levels

**Fig 4:**
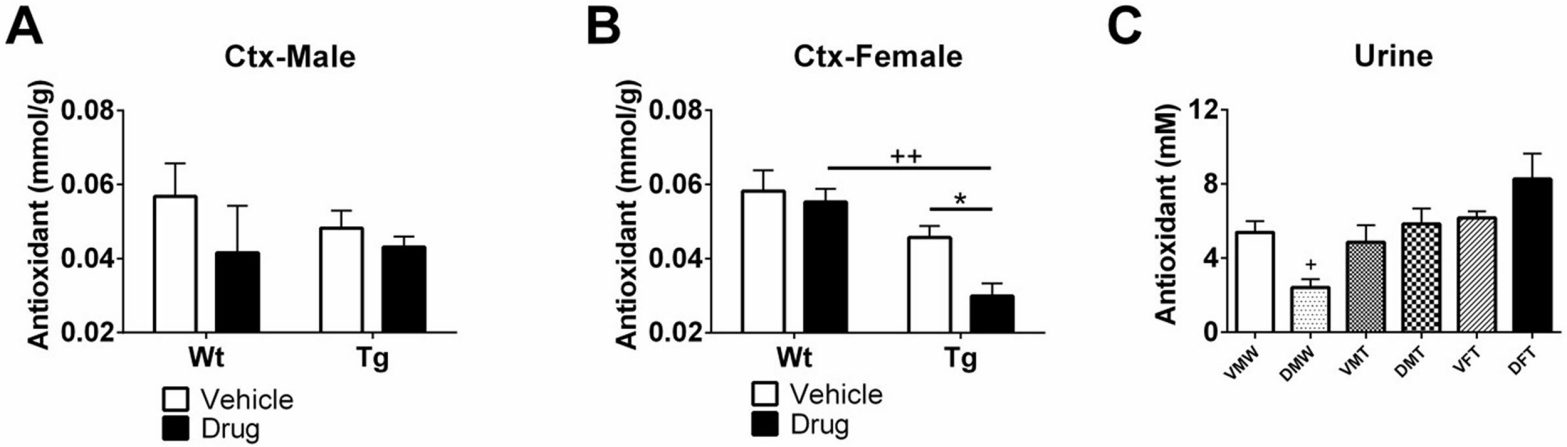
Total antioxidant capacity levels in the cortex of (A) male mice, (B) female mice, and (C) urine samples. VMW: vehicle treated male Wt mice, DMW: drug treated male Wt mice, VMT: vehicle treated male Tg mice, DMT: drug treated male Tg mice, VFT: vehicle treated female Tg mice, DFT: drug treated female Tg mice. Genotype effect (Wt v.s. Tg): +P<0.05, ++P<0.01. Treatment effect (vehicle v.s. drug): *P<0.05. All data are expressed as Mean ± SEM, N = 5 mice/group.

TAC levels as a function of treatment were examined in cortical tissue, serum, and urine. In cortical tissue, no significant difference in TAC was observed in male mice regardless of treatment or genotype (Fig 4A). However, based on genotype, we found a decrease in TAC in female Tg mice treated with the drug compared to Wt mice treated with vehicle (++P<0.01, Fig 4B). In addition, female Tg mice had a prominent reduction in TAC levels compared to vehicle counterparts (*P<0.05, Fig 4B). In terms of genotype effects in urine samples, male Wt mice treated with vehicle had a significant decrease in TAC levels compared to male Tg mice treated with drug, whereas no difference was found between male Tg mice treated with vehicle and male Tg mice treated with drug (+P<0.05, Fig 4C), indicating male Tg might have higher antioxidant capacity levels. No gender differences were observed as a function of R121919 treatment.

**Fig 5:**
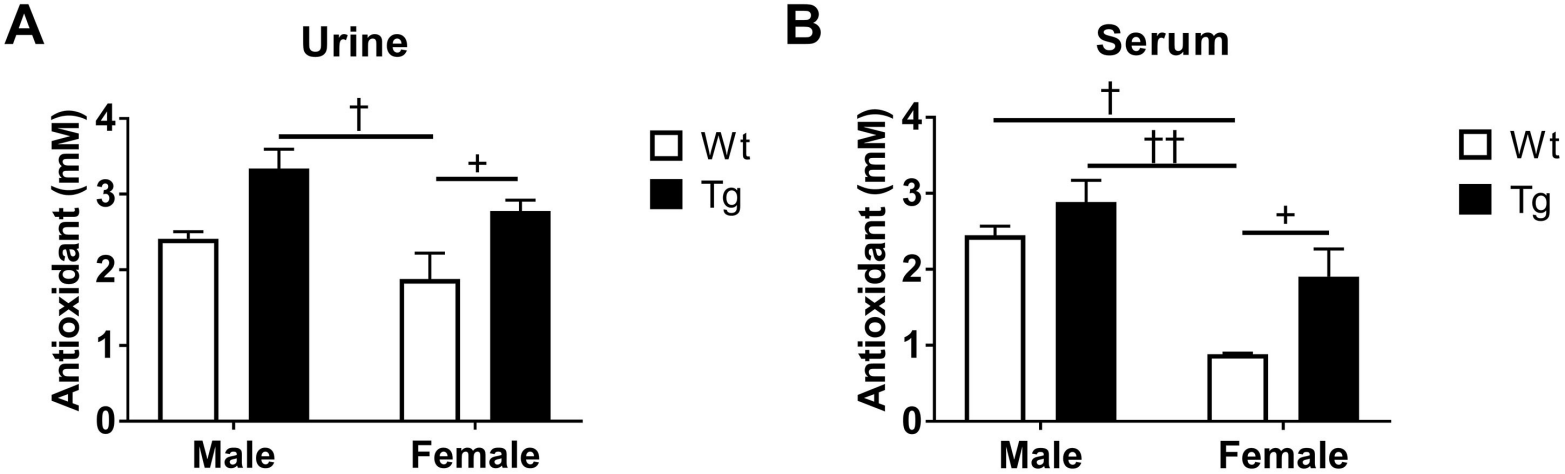
Total antioxidant capacity content in (A) urine and (B) serum samples of male and female mice at 24-month of age. Genotype effect (Wt v.s. Tg): +P<0.05. Gender effect (Male v.s. Female): †P<0.05, ††P<0.01. All data are expressed as Mean ± SEM, N = 5 mice/group.

To compare TAC in aged mice (24-month-old), urine and serum were collected and analyzed. In urine samples, a significant gender difference in TAC was found between male Tg and female Wt mice (†P<0.05, Fig 5A). No genotype effect was found. Nevertheless, an increase in TAC levels was found in female Tg mice compared to Wt counterparts (+P<0.05, Fig 5A). In terms of serum samples, a similar gender effect was seen between male and female samples. For instance, higher levels of TAC were detected in male Wt (†P<0.05) and male Tg (††P<0.01) mice compared to female Wt mice. As for the genotype difference, male mice had no changes in TAC levels, whereas an elevation of TAC levels was observed in female Tg mice compared to the age-matched Wt littermates (+P<0.05, Fig 5B), indicating that female animals might be more sensitive to the changes of TAC levels at this timepoint.

## Discussion

Our previous data demonstrated that R121919 reduces synaptic loss in AD Tg mice and prevent or delay the onset of cognitive impairment [15]. In the present study, we determined whether the beneficial effects of CRFR1 antagonism were linked to modulation of oxidative stress mechanisms.

Our behavioral test for exploratory and anxiety-related behavior revealed no significant differences between the two treatments. In particular, R121919 treated mice had a similar level of locomotor activity as vehicle treated mice during the open-field test. Thereafter, these locomotor and exploratory activities indicate that no abnormal anxiety-like behavior likely related to drug treatment is present. Meanwhile, the animals may have similar motor activity as well as the emotional responses during the test regardless of genotype and treatment differences at this timepoint. This outcome has been reported previously in [20], where locomotor activity in triple-Tg and Wt mice were not different at 6-month-old. This may indicate that no enhanced responsiveness is associated with genotype effect or drug treatment in our mouse model at this endpoint.

### ROS and DNA damage

Studies have implicated oxidative DNA damage in AD neuropathology due to the sensitivity of the brain to ROS. ROS generation is correlated with chromosomal alteration and DNA damage by the processes of hyper or hypomethylation of DNA. Particularly, hypomethylation induced chromosomal changes are whole-arm deletions, isochromosomes, and unbalanced juxtacentromeric translocations, whereas DNA hypomethylation can lead to genomic instability [21]. Only a few studies have explored neuronal stress signaling oxidative stress in AD-like events and we are unaware of any that have assessed this prospectively beginning in the preclinical stage. In this study, therefore, to have a better understanding of the drug effect on levels of ROS and DNA damage, the presence of urine hydrogen peroxide, 8-OHdG, and 5-mdC content was analyzed. As shown in Fig 2, male Tg mice are susceptible to oxidative insults with significantly higher hydroxyl radicals than Wt mice, because the reaction of H_2_O_2_ and metals can generate hydroxyl radicals that are a risk factor of DNA oxidation [22,23]. Moreover, CRFR1 antagonist may have the impact of oxidative stress on reducing hydroxyl radicals. However, H_2_O_2_ levels in the urine of female mice are too low to be detected. One of the possible reasons is that the majority of H_2_O_2_ might react with DNA to lead to elevated 8-OHdG levels in female mice. In addition, female Tg mice treated with vehicle show a significant increase of urine 8-OHdG contents, indicating the antioxidant defense system is inadequate to DNA oxidation. Therefore, DNA lesions are products of DNA modifications induced by oxidative stress, which is commonly caused by alternations of nucleotides. The most reactive free radical is the hydroxyl radical produced by different mechanisms. In particular, the hydroxyl radical from H_2_O_2_ by the Fenton reaction can diffuse in the adjacent nucleus or mitochondrion to attack nDNA or mtDNA [24]. For instance, it reacts with nucleic acid bases directly or indirectly to generated oxidized DNA modifications, such as 8-OHdG and 5-mdC. In addition, 8-OHdG in the frontal and temporal cortices has been reported to be significantly increased in mild cognitive impairment (MCI) patients compared to controls [25,26,27]. Moreover, plasma 8-OHdG levels in transgenic mice [(505 ± 373) pg/mL] are increased compared to Wt mice [(364 ± 287) pg/mL] [28]. We observed a similar genotype effect on urine 8-OHdG levels, where Tg mice treated with vehicle had increased levels compared to their Wt counterparts, suggesting that Tg mice are susceptible to ROS production. Specifically, compared to Tg mice receiving vehicle, there are 91% (*P<0.05, Fig 3A) and 97% (*P<0.05, Fig 3B) decreases in urine 8-OHdG of male Tg and female Tg mice treated with drug, respectively, suggesting that R121919 may mitigate ROS levels, resulting in reducing 8-OHdG contents in AD-Tg mice.

Nevertheless, DNA methylation may silence gene expression that leads to DNA mutation and dysfunction, which is often accompanied with aging in both aged human subjects and experimental animals [29]. A decrease in genomic DNA methylation has been reported in elderly individuals [30]. Likewise, Ray et al. have reported that heterozygous DNA methyltransferase 1 (Dnmt1) deficient mice are susceptible to amyloid pathology with aging. A decrease of global DNA methylation in the brain is correlated with cognitive deficits at 12-month and older age stages compared to 6-month-old mice [31]. It demonstrates that global 5-mdC levels decrease in various tissues and cell lines with normal aging and reduced DNA methylation activity in the brain may promote abnormal methylation modification of the genome in newly formed brain cells to decrease the efficiency in neuron interactions [31]. In this study, the total 5-mdC levels have been analyzed in urine samples of mice under different conditions (Table 2). Analysis of urine 5-mdC concentration revealed that DNA methylation changes are not detectable at this age stage (6-month-old), which is consistent with Liu’s study, where reduced global 5-mdC levels were necessary to extend the endpoint (7-month-old) [31]. Moreover, there was also no significant difference in 5-mdC levels between Wt and Tg mice treated with vehicle, indicating animals might not be prominently susceptible to DNA methylation alternations induced by ROS at this timepoint, and suggesting older animals will be included in the future study. In addition, preconcentration and pretreatment of urine samples will be applied to facilitate the detection of 5-mdC.

### ROS and TAC

Despite deficiencies in vitamins C and E in AD patients compared to healthy controls, studies have demonstrated that plasma TAC levels are not altered, [32]. In this sense, the decline of individual antioxidants may be positively related to the overproduction of oxidative stress, yet TAC levels in animals might remain the same, because of the complexity of the antioxidant system. In this study, we observed a consistent pattern of TAC levels in male AD-Tg mice, indicating no direct relationship with AD neuropathological progression. However, a dramatic increase in H_2_O_2_ level was found in Tg mice treated with vehicle compared to Wt counterparts in Fig 2, and a similar effect can be observed in urine TAC levels in Fig 4C. In addition, significant decreases are observed in female Tg mice treated with drug in Fig 4B, whereas aged female Tg mice (without treatment) have higher TAC levels in both urine and serum samples in Fig 5A and 5B. This might be explained by alteration of free radical activity. Specifically, reduced TAC levels may indicate a low free radical activity and an increase of TAC may be seen as a reflection of elevated free radical activity [33], suggesting that R121919 may have an impact on adjusting TAC levels through mitigating free radical activity.

### The hypothalamic-pituitary-adrenal (HPA) axis and oxidative stress

After corticotropin-releasing factor (CRF) is released from the hypothalamus in the presence of stressful stimuli, adrenocorticotropic hormone (ACTH) from the anterior pituitary gland is released, which signals the release of glucocorticoids from the adrenal cortex. The hypothalamic-pituitary-adrenal (HPA) hypothesis states the correlation between overactivation of HPA axis and oxidative stress, where overproduction of glucocorticoid leads to an increase of oxidative stress, which result in dysregulation of the HPA axis [34]. It has been reported that neurons in the hippocampus are prone to oxidative stress, which leads to overexpression of the HPA axis, resulting in elevated cortisol release in AD neuropathology [13]. Based on these reasons, we investigated the effect of stress signaling blockage on oxidative stress to study whether the levels of hydroxyl radicals and oxidative DNA markers can be modified. Overall, reduced H_2_O_2_ and 8-OHdG in Tg mice treated with R121919 shows that the CRFR1 antagonist may prevent oxidative damage in AD-Tg mice.

Critical mitochondrial functions including mitochondrial calcium capacity, mitochondrial oxidation, and membrane potential can be altered by long-term low-dose treatment of corticosterone, which has a prominent impact on synaptic function and neurotransmitter release and may promote neuroprotection mediated by glucocorticoid and mineralocorticoid receptors [35]. Additionally, Picard’s group has implicated that mitochondrial function might regulate stress response by changing the production of mitochondrial energy, generation of reactive oxygen species and peripheral insulin resistance [36]. Because mitochondrial dysfunction may lead to a reduction of energy supply (ATP) and overproduction of free radicals, alteration of ATP production in mitochondria is a sensitive indicator of brain activity and function and hippocampal-dependent memory can be improved by reducing ROS levels in this study. Based on our previous studies, brain mitochondrial impairment occurred due to ATP decline and mitochondrial ROS increase with aging [37]. A limitation of our current study is our reliance on peripheral biomarkers that are known to reflect changes in the brain, but may be confounded by contribution by organs other than the brain itself. In our future studies, we will investigate the stress response associated with the changes of mitochondrial function, energy production as well as DNA oxidation in the brain.

## Conclusions

Our study demonstrates that a significant increase in urine H_2_O_2_ and 8-OHdG is observed in AD-Tg mice, suggesting that Tg animals are susceptible to hydroxyl radicals, resulting in increasing 8-OHdG content. With chronic treatment of CRFR1 antagonist (R121919) in the preclinical phase of AD, levels of H_2_O_2_ and 8-OHdG are significantly reduced in urine of Tg mice, indicating that R121919 may attenuate oxidative stress by decreasing glucocorticoid in the HPA axis, thereafter, DNA oxidation can be prevented by drug treatment. Collectively, a stress-signaling blockade may be a promising preventative therapy for AD.
